# Newly Isolated *Acidithiobacillus* sp. Ksh From Kashen Copper Ore: Peculiarities of EPS and Colloidal Exopolysaccharide

**DOI:** 10.3389/fmicb.2020.01802

**Published:** 2020-08-05

**Authors:** Narine Vardanyan, Hamlet Badalyan, Levon Markosyan, Arevik Vardanyan, Ruiyong Zhang, Wolfgang Sand

**Affiliations:** ^1^Institute of Microbiology, Scientific and Production Center of “Armbiotechnology” of the National Academy of Sciences of Armenia, Yerevan, Armenia; ^2^Physical Ecology Laboratory, Yerevan State University, Yerevan, Armenia; ^3^Biofilm Centre, Universität Duisburg-Essen, Essen, Germany; ^4^Federal Institute for Geosciences and Natural Resources, Hanover, Germany; ^5^College of Environmental Science and Engineering, Donghua University, Shanghai, China

**Keywords:** iron oxidizing bacteria, bioleaching, copper and copper gold-bearing ore, EPS, colloidal exopolysaccharide

## Abstract

A novel strain of an iron- and sulfur-oxidizing bacterium was isolated from a natural biotope at Kashen copper ore (Martakert Province, Republic of Artsakh). The strain is able to grow and oxidize ferrous ions in the range of pH 1.4–2.6 with optimal pH 2.0. The optimal temperature for growth is 35°C. *Acidithiobacillus* sp. Ksh has shown the highest activity for pyrite oxidation among other strains. It also demonstrated high activity in oxidation for copper and copper-gold bearing ores (Armenia). The isolate *Acidithiobacillus* sp. Ksh was identified as *Acidithiobacillus ferrooxidans* based on phylogenetic and physiological studies. Comparative studies of EPS production by cells grown on ferrous ions or pyrite were carried out. The chemical composition of capsular and colloidal EPS produced by *Acidithiobacillus (At.) ferrooxidans* Ksh were revealed to be proteins and carbohydrates. Exosaccharide produced by *At. ferrooxidans* Ksh is present mainly as polysaccharide in contrast to *Leptospirillum (L.) ferriphilum* CC, which is oligosaccharide. The structural difference of colloidal particles of these polysaccharides was due to the degree of hydration of the saccharide molecules.

## Introduction

Metal bioleaching technology is the use of sulfur- and/or iron-oxidizing bacteria and archaea to extract valuable metals from metal sulfides (MS). The current well-accepted mechanism for MS oxidation is the indirect “contact” leaching (Sand et al., 2001; Vera et al., 2013). Microbial interactions with MS including microbial adhesion on MS and biofilm development are of both fundamental and practical importance (Vera et al., 2013; Li et al., 2020).

Microorganisms usually grow in biofilm lifestyle. Biofilms are a dynamic and 3-D structure of microbial cells embedded in a self-excreted matrix (Costerton et al., 1995; Sutherland, 2001). The matrix represents compounds of extracellular polymeric substances (EPS) which include carbohydrates, proteins, lipids, eDNA, etc. (Flemming et al., 2007; Limoli et al., 2015). Biofilms and EPS mediate adhesion of cells onto substrates or material surfaces, and protect cells from harsh conditions, e.g., desiccation or oxidative stress (Karamanev, 1991; Yu et al., 2008; Bellenberg et al., 2019).

MS oxidation is achieved by ferric ions complexed by EPS:

MS+2Fe→3+M+2+S+02Fe+2

This theory is proven by the fact that the higher amount of ferric ions occurs in EPS of *Acidithiobacillus (At.) ferrooxidans* cells the higher the oxidation rate of pyrite is (Kinzler et al., 2003; Sand and Gehrke, 2006). Thus, MS bioleaching takes place in the biofilm microenvironment – between cells and the MS surfaces filled with EPS. Biofilm formation starts with the adhesion of bacteria to a substrate surface. Adhering to the surface of MS, microorganisms produce EPS, which mediate microbial adhesion on MS and achieve firm attachment (Sand and Gehrke, 2006; Schippers, 2007). It is considered that bacterial recognition of mineral surfaces takes place by chemotaxis (Rodriguez et al., 2003). Sensing the existence of a ferrous ion gradient near the surface mineral, bacteria move along the increase of the gradient. The adhesion is often selective and depends on crystal lattice properties of minerals (Myerson and Kline, 1983; Sanhueza et al., 1999). Thus, it has been revealed that *At. ferrooxidans* selectively adheres to the surface of pyrite (FeS_2_) or chalcopyrite (CuFeS_2_) (Ohmura et al., 1993; Crundwell, 2003; Harneit et al., 2006). It has also been shown that cultures pre-grown on MS attach to pyrite more efficiently than cultures pre-grown on ferrous ions (Africa et al., 2013). Cells of *Sulfobacillus (Sb.) thermosulfidooxidans* pre-grown on pyrite or elemental sulfur prefer to attach to pyrite rather than chalcopyrite (Li et al., 2019).

Another mechanism for biofilm formation is the so-called quorum-sensing system, which implements connection between bacteria by extracellular chemical signals (Rivas et al., 2007; Mamani et al., 2016). EPS are categorized as colloidal (loosely bound) and capsular (tightly bound) compounds (Sheng et al., 2010). Iron and sulfur oxidizing bacteria are able to synthesize both capsular polysaccharides and extracellular colloidal oligo- and polysaccharides (Vardanyan et al., 2019). It has been shown that the EPS composition changes upon growth substrates and growth conditions. EPS of *Sb. thermosulfidooxidans* contain huge amounts of humic acids apart from polysaccharides and proteins (Li and Sand, 2017). Cells of *Leptospirillum (L.) ferrooxidans*^T^, produced increased amounts of total EPS with increasing amount of substrates (galactose or ferrous ions). Cultures pre-grown on galactose produced more sticky EPS than those on ferrous ions (Aguirre et al., 2018). A strategy of overproduction of EPS by a galactose pre-treatment to increase microbial resistance and tolerance to high concentrations of ferric ion and improve the efficiency of *At. ferrooxidans* in biohydrometallurgical processes was proposed (Saavedra et al., 2020). Moderate thermophiles grown on chalcopyrite produced EPS composed of polysaccharides, lipids, proteins and ferric ions (Zeng et al., 2010). Carbohydrates dominated EPS composition of mixed acidophiles in continuously operated bioleaching systems (Govender and Gericke, 2011). EPS of cells of *At. ferrooxidans* R1 contained mostly neutral sugars and lipids (Gehrke et al., 1998). EPS production is species and strain specific. Detailed analyses of EPS produced by extremophiles, e.g., acidophilic bioleaching microorganisms, are still largely missing (Flemming and Wingender, 2010; Zhang et al., 2016).

The aim of this work was to study the chemical composition of EPS plus formation and characteristics of exopolysaccharides excreted by a new iron-oxidizing bacterium *Acidithiobacillus* sp. Ksh.

## Materials and Methods

### Strains and Cultivation

The strain of an iron-oxidizing chemolithotrophic bacterium Ksh was isolated from the natural biotope of Kashen copper ore (Martakert Province, Republic of Artsakh). The pH of sampling site was about 2.5 and temperature 15°C. To obtain an enrichment culture of iron- and sulfur-oxidizing bacteria, Mackintosh (Mac) medium (Mackintosh, 1978) containing 4.9 g/L ferrous ions as a source of energy was inoculated with acid mine drainage water and cultivated at 30°C and 37°C for 1–2 weeks.

The enrichment culture was used to isolate a pure culture of iron-oxidizing bacteria. The Manning (Manning, 1975) solid medium was used to obtain single colonies, which were transferred to the liquid Mac medium for further identification.

### Microscopic Studies

Gram staining was performed by the Hooker method (Gerhardt et al., 1981). Cell morphology was studied with light microscope (Leica DM500) and by scanning electron microscopy.

### Optimal pH and Temperature for Growth

To determine the optimal temperature for growth the iron-oxidizing activity of cells was assayed from 25 to 45°C in Mac medium. The optimal pH for iron oxidizing activity was studied in the range of pH from 1.1 to 2.9 (HANNA Instruments Inc., United Kingdom). The maximum specific growth rate (μ_*max*_) was determined using the Monod equation:

μ=2.303(lgN-lgN)0/t-t0

where *N* is number of cells, *t* is time. The doubling time (*g*) was determined by the equation:

g=0.693/μ

### Strain Identification

DNA was extracted using the protocol previously reported (Aljanabi and Martinez, 1997). 16S rRNA amplification was conducted according to the method previously described (Vardanyan et al., 2017). Extraction and purification of the PCR-product from the agarose gel were done using Zymoclean^TM^ Gel DNA Recovery Kit (ZYMO RESEARCH). The purified PCR products of approximately 1400 bp were used for sequencing (MACROGEN, South Korea).

Releated 16S rRNA genes were downloaded from NCBI and were compared online by BLAST (NCBI). The phylogenetic trees were constructed by using MEGA (version 6.06) and Neighbor-joining method.

### Bioleaching of Metal Sulfides

Selected strains were grown on Mac medium containing Ferrous ions as substrate (Johnson et al., 2005). Cells in logarithmic phase were harvested by centrifugation (8000 rpm, 10 min), washed with acidified Mac medium for further use. Pyrite (FeS_2_), or copper ore (sulfide) containing 23.9% Cu, 29.2% Fe, 39.6% S (Martakert Province, Republic of Artsakh) both ground to 45–63 μm were placed into 250 mL Erlenmeyer flasks containing 50 mL of Mac medium. Initial pH and cell concentration were 2.0 and ∼10^8^ cells/mL. Bioleaching assays were carried out at 30 or 35°C and shaking (180 rpm/min). Sampling was performed at 24 h intervals. pH, dissolved ferric and ferrous ions in the medium were analyzed by titration with EDTA. Total concentrations of copper and iron were measured by atomic absorption spectrometry (AAS 1N, Germany). The experiments were performed in triplicate. The data were analyzed statistically by Excel using student *t*-test.

### EPS Extraction

Cells of *Acidithiobacillus* sp. Ksh grown in Mac medium (4.9 g/L ferrous ions or 10% pyrite) at 35°C were collected by centrifugation (8000 rpm, 10 min) at room temperature, washed and resuspended in 10 mL sterile Mac medium for EPS extraction as previously described (Castro et al., 2014). Ferric ions in the extracted EPS solution were precipitated by adding NaOH till pH 7.5–8.0. The precipitates were removed by centrifugation and the concentration of the supernatant was performed in a rotary evaporator at 40°C to 20% of the initial volume, the polysaccharides were separated using the method previously described (Markosyan et al., 2019).

### Determination of EPS Composition

Carbohydrate content was determined by the protocol of Dubois (Dubois et al., 1956). The content of protein was determined according to Bradford (1976). Uronic acids were quantified by the protocol of Blumenkrantz and Asboe-Hansen (1973). The content of proteins, carbohydrates, and uronic acids in EPS areexpressed in μg/mL culture medium. The exopolysaccharide was hydrolyzed in 2 M HCl at 100°C for 2 h and then analyzed by HPLC (on the Shimadzu 2010 C analyzer, column Ultron PS-80-H, 2 × 250 mm, mobile phase 0.1 mM acetate buffer/accetoniteile 1:5, pH 5.8, gradient flow rate 1–0 mL/min) according to the protocol previously described (Markosyan et al., 2019). Measurements were performed by Refratometric Index (RI). For elucidation of the changes in the size of colloidal formations, the degree of their hydration was studied using analytical program Lab VIEW*-*15 and WISION (Badalyan and Astanyan, 2016; Margaryan et al., 2016). The results were transformed in accordance with the NOVA3.5PC program.

## Results and Discussion

### Strain Characterization and Identification

The new iron- and sulfur- oxidizing bacterium Ksh was obtained from the natural biotope (acid mine drainage) of Kashen copper ore. It has been deposited in the Republican Centre for Deposition of Microorganisms of the National Academy of Sciences and Ministry of Education and Science of Armenia under the number MDC7056. Cells are Gram-negative and motile, often occurring as single cells, if grown on elemental sulfur, ferrous ions or pyrite. As shown in Supplementary Figure S1, cells are rod-shaped with a length of 0.7–2 μm.

**FIGURE 1:**
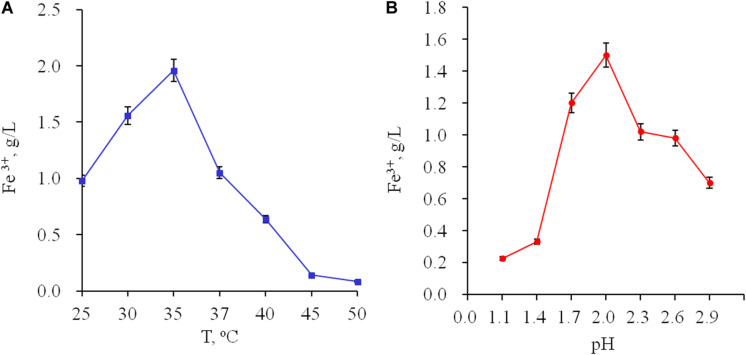
Influence of temperature **(A)** and pH **(B)** on oxidation of ferrous ions by *Acidithiobacillus* sp. Ksh.

The main physiological properties were studied. The optimal temperature for growth was 35°C (Figure 1A). Growth of the strain on ferrous ions is possible in the range of pH 1.1–2.9 with an optimal pH 2.0 (Figure 1B). *At. ferrooxidans* Ksh showed autotrophy and its growth was inhibited in the presence of yeast extract (data not shown). The maximum specific growth value was 0.38 h^–1^ at an ferrous ion concentration of 100 mM. Doubling time for the growth of *At. ferrooxidans* Ksh was 1.8 h.

The 16S rRNA gene sequence of *Acidithiobacillus* sp. Ksh was deposited in GenBank (No. MN539150). Phylogenetic analysis showed that *Acidithiobacillus* sp. Ksh is affiliated to *At. ferrooxidans* (Supplementary Figure S2). The strain shares similar properties in morphology and physiology with the *At. ferrooxidans* type strain ATCC 23270. However, certain differences between strains *Acidithiobacillus* sp. Ksh and the type strain exist. e.g., optimum growth temperature of *Acidithiobacillus* sp. Ksh is 35°C and of the type strain 30°C.

### Bioleaching of Ores

**FIGURE 2:**
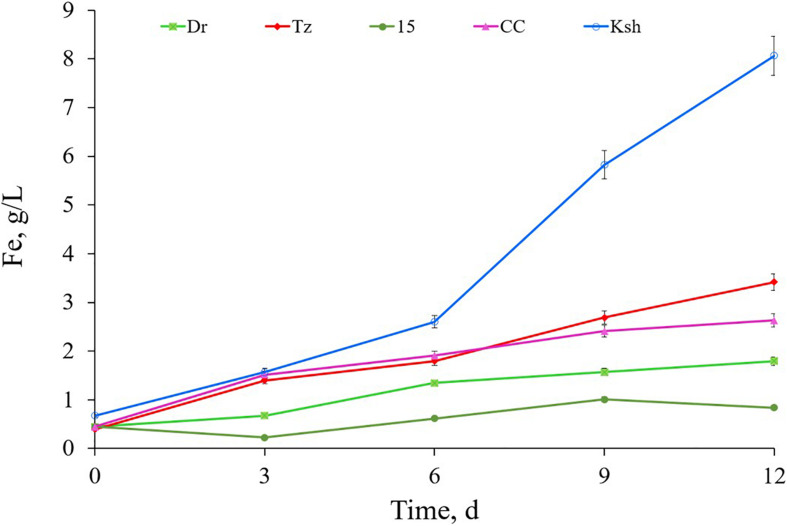
Bioleaching of pyrite by isolated iron-oxidizing bacteria *Acidithiobacillus* sp. Tz, Dr, 15, Ksh and *Leptospirillum* sp. CC (pulp density 4%, temperature 30°C, pH 2).

Comparative activities of *Acidithiobacillus* sp. Ksh and that of the type strain of iron-oxidizing bacteria (Vardanyan et al., 2015) for pyrite oxidation are presented in Figure 2. As seen from the data, *Acidithiobacillus* sp. Tz and *L. ferriphilum* CC showed similar activities for pyrite oxidation. *Acidithiobacillus* sp. Dr and *Acidithiobacillus* sp. 15 were inferior to the above-mentioned strains. The new isolated strain *At. ferooxidans* Ksh had the highest activity for pyrite oxidation. Its pyrite oxidation activity exceeded those of *Acidithiobacillus* sp. Tz and *L. ferriphilum* CC about 2.1–2.3 and 2.4–3.0 times, respectively (Figure 2). *At. ferrooxidans* Ksh accelerated the oxidation of pyrite by approximately 5.3 times over an abiotic oxidation at 10% pulp density (PD) (Supplementary Figure S3).

**FIGURE 3:**
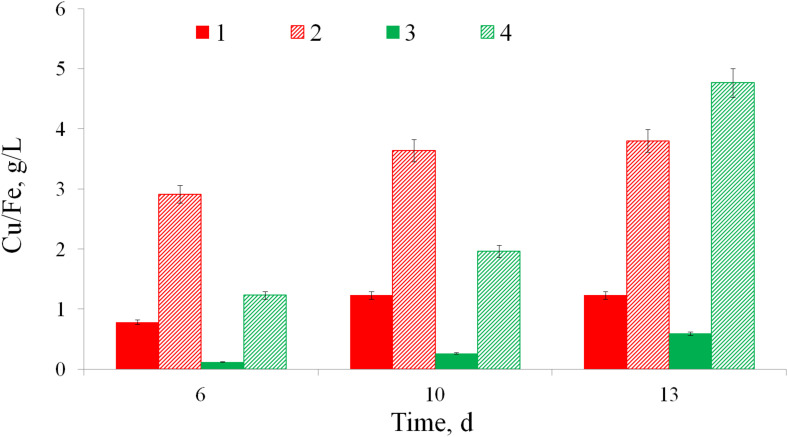
Bioleaching of iron (1, 2) and copper (3, 4) from a copper ore without bacteria (1, 3) and by *At. ferrooxidans* Ksh (2, 4) (pulp density 10%, temperature 35°C).

Biooxidation of copper ore by *At. ferrooxidans* Ksh is presented in Figure 3. Data indicate that extraction of copper and iron from copper ore by *At. ferrooxidans* Ksh increased about 7–8 and 3 times, respectively, in comparison with the abiotic oxidation.

### EPS Analysis

As shown in Table 1, the total EPS amounted to 38 μg/L for cells grown on ferrous ions. In contrast, pyrite-grown cells produced 2335 μg/L EPS. Thus, cells of *At. ferrooxidans* Ksh excrete much more EPS when cultivated on pyrite, if compared to cells cultivated on ferrous ions. This phenomenon is in agreement with the previous reports (Gehrke et al., 1998; Vardanyan et al., 2019).

**TABLE 1:** EPS composition (μg/L) of *At. ferrooxidans* Ksh grown on ferrous ions and pyrite.

EPS composition		Energy substrates	
		Ferrous ions	Pyrite
Colloidal	Protein	17	605
	Carbohydrates	6	420
	Uronic acids	BDL*	BDL
Capsular	Protein	9	760
	Carbohydrates	6	550
	Uronic acids	BDL	BDL

*BDL: below the detection limit.

Table 1 shows that the amount of carbohydrates in capsular EPS when cells were cultivated on ferrous ions was considerably greater than that in colloidal EPS. By contrast, colloidal EPS contained higher amounts of protein in comparison with capsular EPS. When cultivated on pyrite, cells generally produced higher amounts of protein than those of carbohydrates, for both capsular and colloidal fractions. Uronic acid in both cases was not detected or below the detection limit (BDL).

### Monosaccharides

**FIGURE 4:**
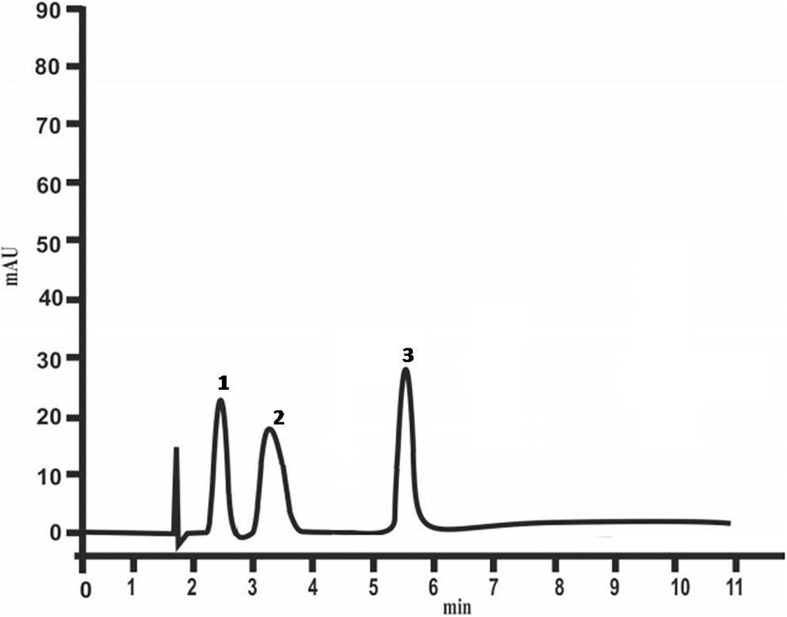
Chemical composition of colloidal polysaccharides of *At. ferrooxidans* Ksh (1 – glucose, 2 – fructose, 3 – xylose) analyzed by HPLC.

HPLC studies have revealed the presence of the following sugars: glucose, fructose and xylose in colloidal exopolysaccharides (Figure 4). Glucose and xylose had been detected also in the EPS of *At. ferrooxidans* R1 (Gehrke et al., 1998).

**FIGURE 5:**
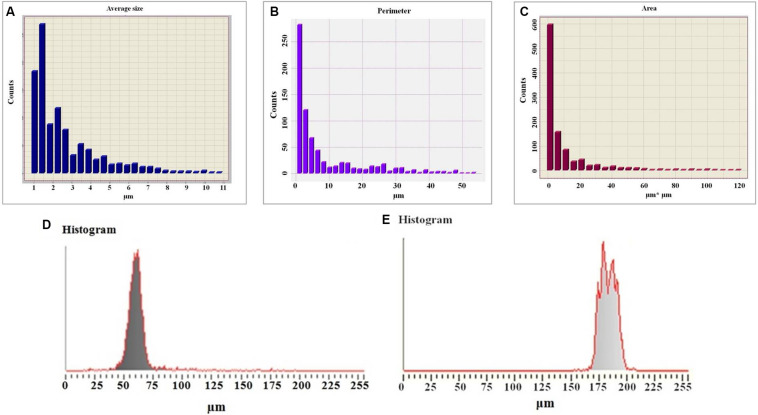
Histogram of the average sizes of colloidal particles of exopolysaccharide **(A)**, histogram of the perimeters of colloidal particles of a polysaccharide **(B)**, histogram of the area of colloidal particles of a polysaccharide **(C)**, histogram of the micrometry of colloidal particles of exopolysaccharides **(D)**, histogram of the micrometry of colloidal particles of a diluted solution of a polysaccharide **(E)**.

Study of colloidal particles of exopolysaccharides by optical polarized microscopy indicate that they have high degree of crystallization. This is also shown by micrometric studies of optical images of colloidal particles (Figure 5). The histogram shows that oligosaccharides have a high degree of crystallization. The size of colloidal particles of the exopolysaccharides changes exponentially starting with 10 μm (Figure 5A), while in case of *L. ferriphilum* CC it does not change regularly (Markosyan et al., 2019). The perimeters and area of these particles also change exponentially (Figures 5B,C), ensuring the stability of their shape parameters determined by a = S/p^2^, S is the area, P is the perimeter.

The studies also reveal that the degree of crystallization of colloidal particles (Figure 5E) decreased with dilution (five times) of the polysaccharide solution. This is obvious from the results of micrometry of colloidal formations (Figure 5A). Therefore, the half width of the histogram is almost 1.5 times greater (28.5 μm) than before dilution. There is no clear maximum indicating that the degree of crystallization of colloidal formation decreased (Figure 5D). This may be due to the fact that in parallel with the decrease in the perimeters of colloidal particles the degree of their hydration also decreases.

**FIGURE 6:**
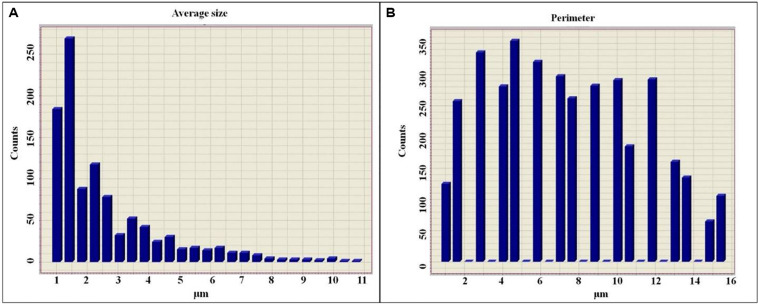
Histogram of the sizes of colloidal particles of diluted solutions of exopolysaccharides **(A)**, histogram of the perimeters of colloidal particles of diluted solutions of exopolysaccharides **(B)**.

The histogram of the average size of colloidal particles is represented as an exponentially reduced function (Figure 6A), whereas the histogram of the perimeter is closer to a Gaussian distribution (Figure 6B), which in turn is a consequence of the different degree of hydration. It is also shown that in parallel with the reduction of the perimeters of colloidal particles, their degree of hydration also decreases.

**FIGURE 7:**
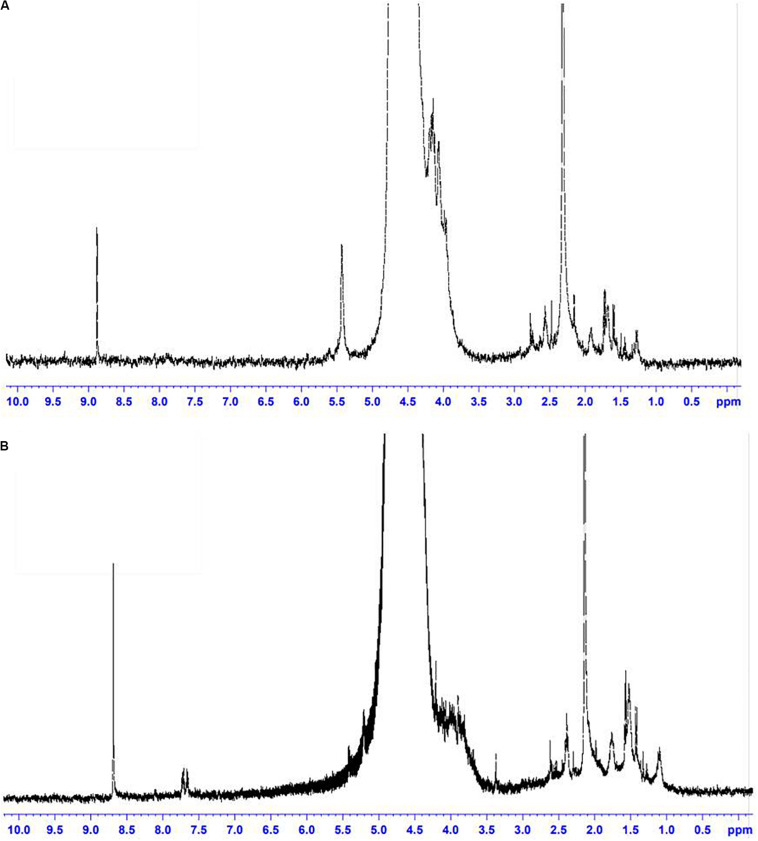
NMR spectra: **(A)** oligosaccharide produced *by L. ferriphilum* CC, **(B)** exopolysaccharide produced by *At. ferrooxidans* Ksh.

**FIGURE 8:**
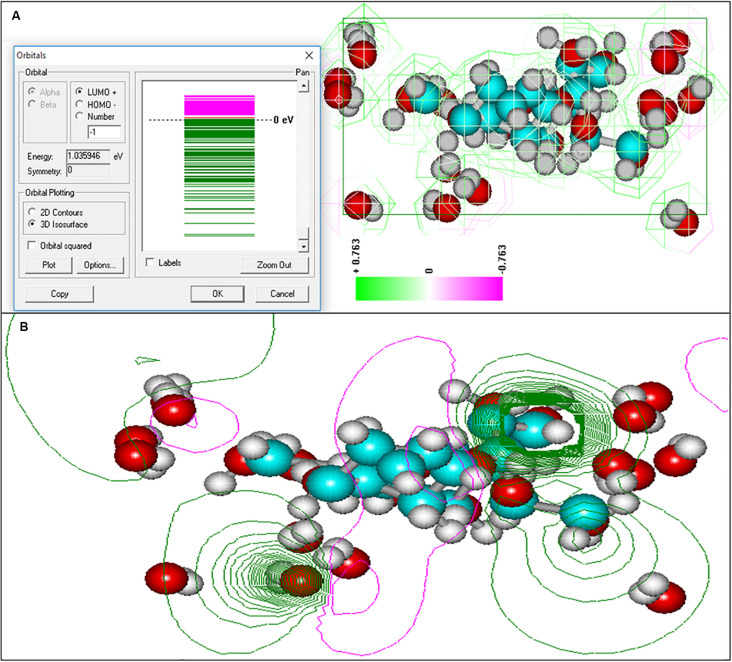
Electronic spectra of oligosaccharide and equipotential of the surfaces of colloidal formation of *At. ferrooxidans* Ksh obtained by semi-empirical quantum modeling with 3D option **(A)**, 2D option **(B)**.

**FIGURE 9:**
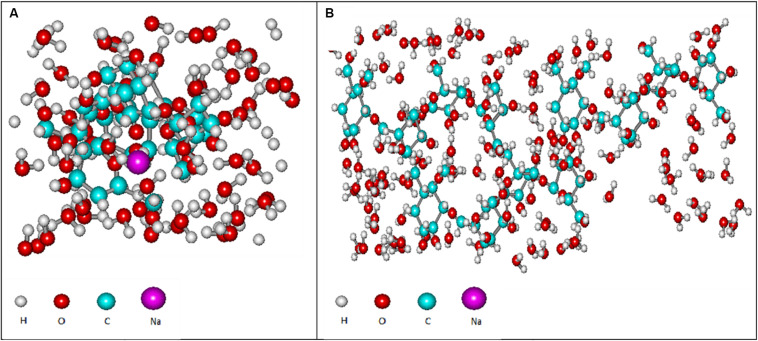
Computer models of the hydrated polysaccharide of *At. ferooxidans* Ksh in the presence of salt **(A)** and without salt **(B)**, obtained by using semi-empirical and quantum mechanical modeling.

Based on the experimental data, it can be assumed that there are differences between the size and structure of the colloidal particles of polysaccharides produced by different microorganisms (Markosyan et al., 2019). To clarify the reasons for the differences in the sizes of colloidal particles, NMR studies of exosaccharides produced by *L. ferriphilum* CC and *At. ferrooxidans* Ksh were performed (with *D*_2_*O*, 1H with BRUCER, Avance 400 Neo TopSpin 4.0, Latvia). The degree of hydration of the oligosaccharide of *L. ferriphilum* CC in the colloidal formation detected by NMR was studied. The data indicate structural differences for colloidal exopolysaccharides produced by these strains (Figures 7A,B). The differences are caused by the fact that the exosaccharide produced by *L. ferriphilum* CC is mainly an oligosaccharide, while that of Ksh is mainly a polysaccharide and depends on cultivation conditions. It was revealed that hydration of colloidal particles, especially its high level, was caused by sodium ions located in the center of colloidal particles resulting from the dissociation of salt contained in the medium for the isolation of polysaccharides (Figures 8, 9).

Studies have shown that the hydration of the exopolysaccharides in colloidal formation occurs in three layers. The first layer closest to an oligosaccharide molecule has a pentagonal structure and resembles ice of the second type (Badalyan and Grigoryan, 2006).

As shown in Figure 9, the next hydration layer is connected by hydrogen bonds with the first layer, and depends on the thickness of the water layer, gradually and subsequently acquires the structure of free water. The third hydration layer is represented by free water and does not participate in the formation of colloidal particles. For this reason the increase in concentration of oligosaccharide produced by *L. ferriphilum* CC does not lead to an increase of the particle size, but rather contributes to the formation of new, identical particles. This is in contrast to the polysaccharide produced by Ksh. Ions resulting from the dissociation of salt in the solution change only the conformation of the polysaccharide molecule. In this case a low level of hydration of saccharide is observed. An increase in polysaccharide concentration (0.07%) does not lead to an increase in the number of particles, but contributes to an increase in their size. In order to elucidate the changes caused by three-layer hydration of saccharides in colloidal formations, the equipotential surfaces of electric fields as well as the electronic spectra of these oligosaccharide particles were studied by computer modeling using the indicated programs. Studies of the equipotential surfaces of colloidal formations (according to the 3D option) showed that the lines were concentrated around the oligosaccharide molecule and easily interacted with molecules of surrounding water or ions (Figures 8A,B). As a result, water molecules located close to the surface of the saccharides bind strongly to them, forming the so-called “frozen structures” (Badalyan and Grigoryan, 2006). The electronic states of the oligosaccharide molecule shown in Figure 9A according to 3D option also contribute to this. Similar studies of diluted solutions (0.07%) showed that the equipotential surfaces of the electric fields of colloidal formations, although relatively not dense (Figure 9B), extended to relatively large distances from the molecule. As a result, they easily bind to salt ions, providing a relatively high level of hydration. Due to this effect of salt ions the increase of colloidal particles occurs.

Thus, the formation and properties of colloidal particles of these saccharides are significantly different. In the case of an oligosaccharide the increase in its concentration does not lead to an increase in the size of colloidal particles but contributes to the formation of new identical particles. Besides, the degree of hydration of oligosaccharide is much higher compared with the polysaccharide, which in turn determines the nature of the formation of colloidal particles. It was also shown that the hydration of saccharide molecules occurred in three layers and a significant role is played by the salt ions in the medium.

## Conclusion and Significance

The newly isolated *At. ferrooxidans* Ksh strain from a natural biotope at Kashen copper-molybdenum ore (Armenia) demonstrated high activity for oxidation of copper and copper-gold bearing ores. The extraction of copper and iron from a copper ore by *At. ferrooxidans* Ksh reached 4.8 and 3.6 g/L respectively. Further studies have shown that the cells of *At. ferrooxidans* Ksh produce much higher amounts of total EPS, if grown on pyrite, compared to the ones grown on ferrous ions.

The capsular EPS contain higher amounts of carbohydrates, while colloidal EPS contain more protein compared with capsular EPS. In case of pyrite as substrate, the amount of protein is generally higher than that of carbohydrates for both, capsular and colloidal EPS. Uronic acids in both cases were not observed.

Exosaccharide produced by *At. ferrooxidans* Ksh is present mainly as polysaccharide in contrast to *L*. *ferriphilum* CC, which is an oligosaccharide. In case of the polysaccharide of *At. ferrooxidans* Ksh an increase in concentration leads to an increase in size. It was revealed that the structural difference of colloidal particles of the mentioned oligosaccharide and polysaccharide was due to the degree of hydration of their molecules.

## Data Availability Statement

The datasets generated for this study can be found in the repositories. The names of the repository/repositories and accession number(s) can be found in the article/Supplementary Material.

## Author Contributions

NV and RZ conceived and designed the experiments. HB, LM, and AV performed the experiments. NV, AV, and HB analyzed the data. NV and WS contributed to the research materials. NV and AV wrote the manuscript. RZ and WS have overseen all aspects of this project in terms of scientific significance. All authors contributed to the article and approved the submitted version.

## Conflict of Interest

The authors declare that the research was conducted in the absence of any commercial or financial relationships that could be construed as a potential conflict of interest.
