# Supplementary material for: Newly Isolated Acidithiobacillus sp. Ksh From Kashen Copper Ore: Peculiarities of EPS and Colloidal Exopolysaccharide
Source: Front Microbiol. 2020 Aug 5;11:1802. doi: 10.3389/fmicb.2020.01802 (PMC7419681; doi:10.3389/fmicb.2020.01802)
Supplement: Supplementary file 1 [file Data_Sheet_1.docx]

**SUPPLEMENTARY MATERIAL**


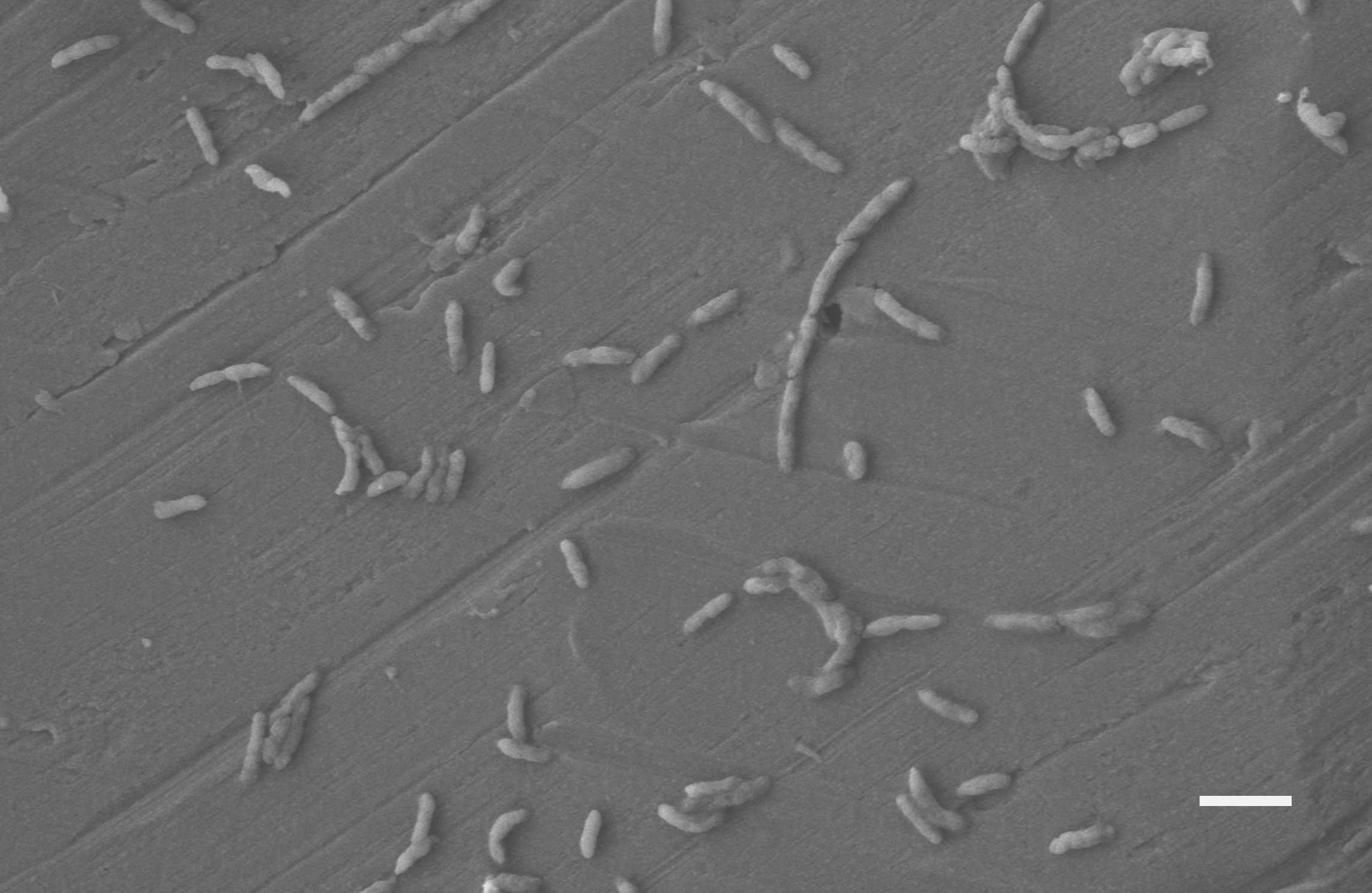


**Supplementary Fig. S1.** Cell morphology of the isolate Ksh by SEM. Bar represents 2 µm


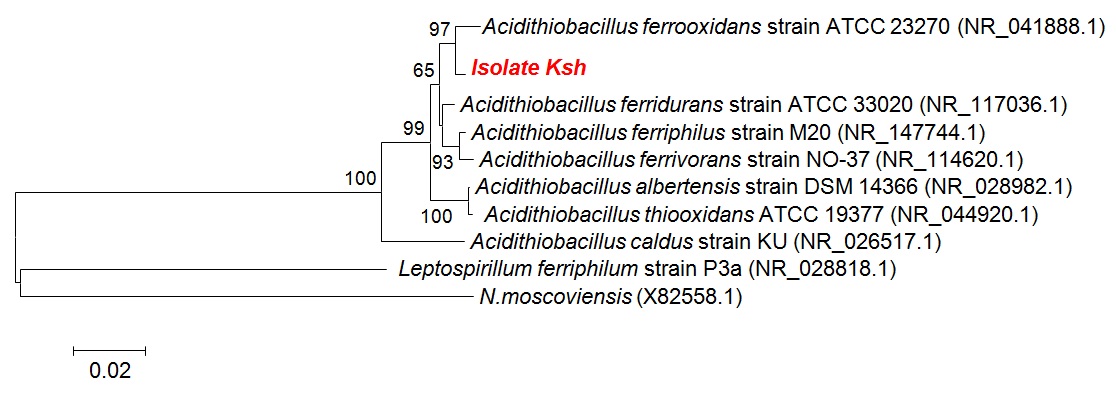
**Supplementary Fig. S2.** Phylogenetic tree of strain *Acidithiobacillus* sp. Ksh. The phylogenetic trees were constructed by using MEGA (version 6.06) and Neighbor-joining method. Bootstrap analysis was carried out on 1000 replicate input data sets.


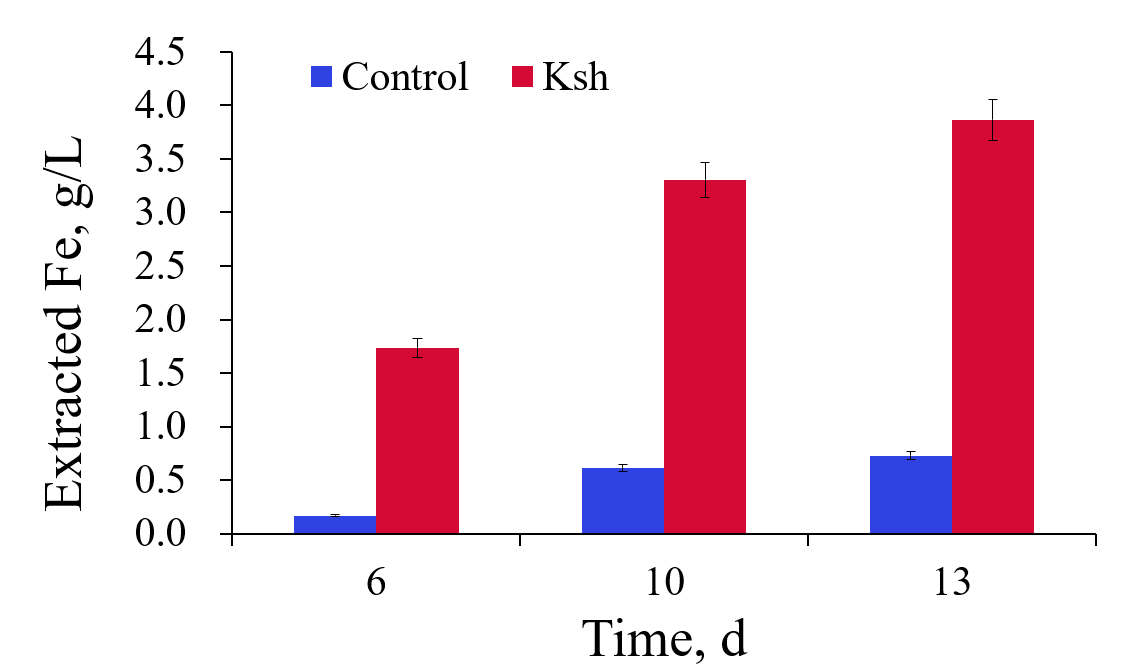


**Supplementary Fig. S3.** Bioleaching of pyrite by cells of *At. ferrooxidans* Ksh

(PD 10%, t 35 °C)
